# Tenon’s patch graft to the rescue during COVID-19 pandemic

**DOI:** 10.3205/oc000218

**Published:** 2023-03-01

**Authors:** Suchitra Panigrahi, Bidisha Mahapatra, Swarnamayee Baskey

**Affiliations:** 1 Department of Ophthalmology, Pandit Raghunath Murmu Medical College, Baripada, India

**Keywords:** Tenon’s graft, Mooren’s ulcer, COVID-19, amniotic membrane

## Abstract

**Objective:**

To report a case of bilateral Mooren’s ulcer with impending corneal perforation in the right eye and perforated peripheral corneal ulcer in the left eye which successfully underwent Tenon’s patch graft (TPG) with multilayered amniotic membrane grafting (AMG) as globe salvaging procedure during COVID-19 pandemic.

**Methods:**

Conjunctival resection was done on both the eyes followed by autologous Tenon’s grafting with overlay amniotic membrane grafting in the left eye with perforation and multilayered AMG with AMG overlay in the right eye with impending perforation. Post-operatively topical antibiotics and steroids were prescribed.

**Results:**

At one month follow-up, the Tenon’s graft and multilayered AMG were well-integrated with the surrounding healthy cornea with a good anatomical and visual outcome.

**Conclusion:**

Autologous Tenon’s patch graft is a simple and cost-effective alternative to preserve globe integrity and prevent complications in emergency cases when immediate access to cornea transplantation is practically challenging.

## Introduction

Corneal perforations may result from a variety of inflammatory or infectious causes. Mooren’s corneal ulcer, first described by Bowman in 1849 [1], is one such rare cause, in which antibodies are directed against corneal stroma [2], leading to peripheral corneal ulceration, thinning and perforation. If not treated urgently, it can lead to complications such as phthisis bulbi, hypotony, peripheral anterior synechiae formation, endophthalmitis or panophthalmitis [3]. The various options for management of corneal perforation described so far include tissue adhesives with bandage contact lenses, multilayered amniotic membrane grafting (AMG), corneal patch graft and penetrating keratoplasty. However, the ongoing SARS-CoV-2 (COVID-19) pandemic has imposed unprecedented restrictions in eye banking throughout the world, resulting in extreme donor corneal tissue shortage even in the tertiary eye care centres [4]. Moreover, due to interruption in transport facilities due to lockdown, referral of patients from centres with no eye banking facility like ours to higher centres for keratoplasty has also been affected. In such a scenario, we report a case of bilateral peripheral ulcerative keratitis that successfully underwent autologous Tenon’s patch grafting with multilayered AMG in our hospital as globe salvaging procedure during COVID-19 pandemic.

## Case description

A 47-year-old male from a rural tribal area with low socio-economical status presented to us with the complaints of decreased vision, redness, watering, ocular pain and photophobia in both eyes for the last one month, more so in the left eye for the last one week. There was no history of trauma or associated joint pain. 

**Figure 1:**
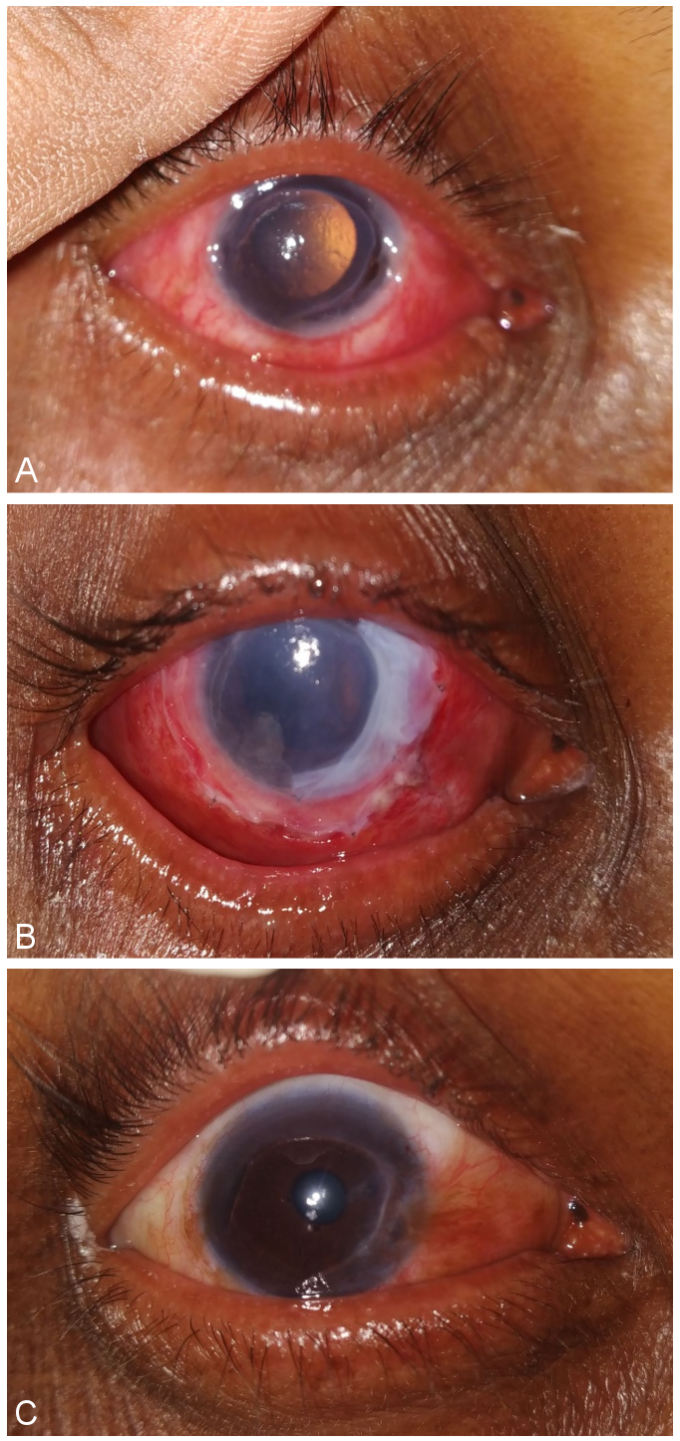
(A) Pre-operative picture of RE showing Mooren’s ulcer with impending perforation; (B) post-operative day 1; (C) post-operative 1 month

**Figure 2:**
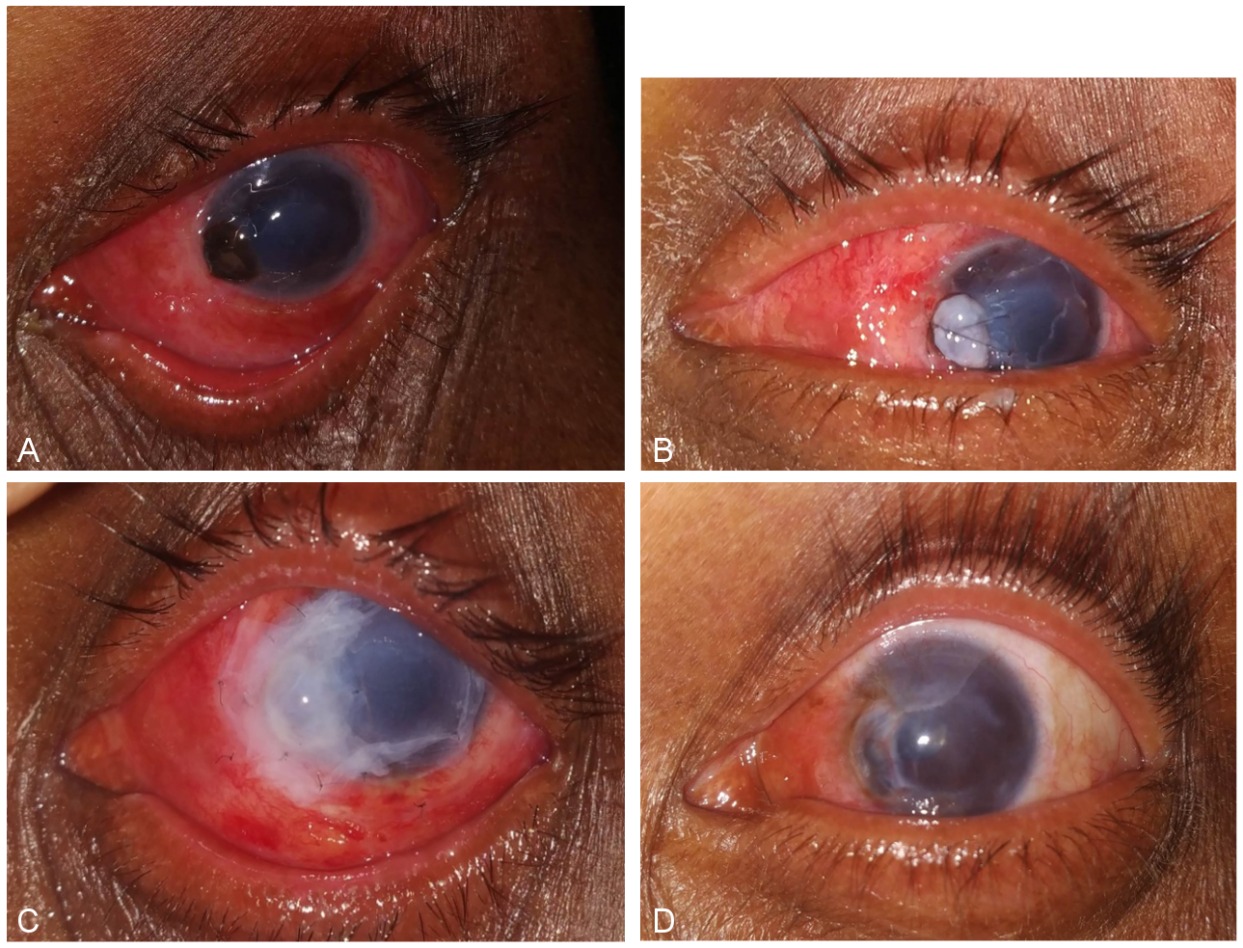
(A) Preoperative picture of LE showing peripheral corneal perforation with iris prolapse; (B) Tenon’s patch graft anchored to recipient bed with cross suture; (C) post-operative day 1; (D) post-operative 1 month

On examination, best-corrected visual acuity in the right eye was 6/60 and in the left eye was counting finger at 1 metre distance. In the right eye, there was peripheral corneal thinning involving 6 clock hours (12–6 o’clock) with limbal involvement without associated scleritis (Figure 1A (Fig. 1)). In the left eye, there was peripheral corneal perforation measuring 3 mm*2.5 mm with iris prolapse at 8–9 o’clock position (Figure 2A (Fig. 2)). In both eyes, the ulcer was characterized by overhanging edge spreading centrally and circumferentially. Fluorescein staining in the left eye showed positive Seidels with adjacent 6 mm*5 mm corneal epithelial defect. The pupil was peaked nasally due to perforation. There were no signs of secondary infection and/or iritis.

Investigations including hemogram with ESR, RBS, urine routine and microscopy, VDRL, RA factor, HCV, X-ray chest and joints, SGPT, ANCA, ANA, and HBsAg were done. Scraping from the ulcer did not reveal any causative organisms on smear or culture. After excluding systemic diseases associated with peripheral ulcerative keratitis by a physician based on the investigations done and clinical examination, a diagnosis of bilateral Mooren’s ulcer was made and systemic immunosuppressive therapy in the form of intravenous prednisolone 1 mg/kg body weight was started. 

In the right eye conjunctival resection under local anaesthesia was done, 2 clock hours on either side of the ulcer and 4 mm posterior to corneoscleral limbus. Debris and loose epithelium over the area of severe thinning was removed, and the crater was filled with multiple (five) layers of freshly prepared amniotic membrane fashioned approximately to the size of the crater. A second amniotic membrane was transplanted as a basement membrane with the epithelial side up and secured with 10-0 nylon sutures. Finally, a larger piece of AM with the epithelial side up was applied over the entire cornea as a temporary patch and anchored with 10-0 nylon sutures to the corneal limbus and perilimbal episclera. 

In the left eye, conjunctival resection was done similar to the left eye. The recipient bed was prepared by removing all the debris over the perforation, 2 mm of epithelium around the perforation was removed with a 26-G needle and the bed around the perforation was dried with a Weck-Cell sponge. Superficial conjunctiva was incised 2 mm away from the limbus in the inferotemporal quadrant and Tenon’s of the size slightly larger than the size of corneal perforation was harvested. The harvested Tenon’s graft was ironed into a thin layer, placed over the perforation and anchored to the host bed by placing an overlay cross suture using 10-0 nylon (Figure 2B (Fig. 2)). The graft was covered by two layers of amniotic membrane similar to the right eye for the growth of healthy epithelium and integration of the Tenon’s tissue with the surrounding healthy corneal stromal bed. Air was injected into the anterior chamber, iris tissue adherent to the graft was gently separated, anterior chamber was formed by reinjecting air bubble and side port was hydrated. 

Postoperatively, the patient was prescribed eyedrop prednisolone acetate 1% two-hourly, moxifloxacin hydrochloride 0.5% four times a day, homatropine hydrobromide 2% thrice a day and carboxymethylcellulose 1% six times a day. On post-operative day 1, the Tenon’s patch graft (TPG) in the left eye was well-attached and the anterior chamber was formed with no leak on Seidel test (Figure 2C (Fig. 2)). Multilayered AMG in the left eye was well-attached (Figure 1B (Fig. 1)).

On one week follow-up, the big overlay AMG was loose and overhanging and was, therefore, removed. Corneal epithelium had healed completely and Tenon’s graft was well-apposed in the left eye. In the right eye, the stromal melt was well-stabilized and the AMG layers were well-integrated with the host tissue.

On one month follow-up, BCVA had improved to 6/18 in the right eye (Figure 1C (Fig. 1)) and 6/60 in the left eye (Figure 2D (Fig. 2)) with immature cataract in both the eyes. No recurrence was noted.

## Discussion

The use of Tenon’s capsule has been described in the management of traumatic scleral perforations and post-trabeculectomy leaking blebs [5]. Recently the use of TPG in conjunction with cyanoacrylate glue has been described in the management of large corneal perforations, measuring 3–6 mm, where tissue adhesive alone is not suitable [6]. Sharma et al. [7] described tuck in TPG technique in cases of sterile corneal perforations of size 3–5 mm. Maharana et al. [8] used TPG without glue to manage a rare case of corneal fistula. Most of the cases reported earlier were central corneal perforations unlike ours which was a peripheral corneal perforation. Tenon’s being an opaque tissue when used in central corneal perforation due to scar formation leads to suboptimal visual recovery which is not much of a concern in peripheral perforations. The success of TPG has been reported to be 75%, by Korah et al. [6] and 82.7%, as reported by Sharma et al. [7]. Tissue adhesives are not only costly but also lead to increased postoperative inflammation, vascularisation and ocular discomfort in cases of corneal perforation. Whereas, Tenon’s being an autologous tissue, evokes no immune response thus eliminating any chances of tissue rejection making corneal grafting more likely to succeed if performed at a later stage. Further, unlike corneal grafting or AMT, this tissue does not rely on donor tissue availability or eye bank facility making it a cost-effective technique in developing countries.

## Conclusion

Autologous nature of the tissue, minimal chances of rejection, easy availability, cost-effectiveness and a simple surgical technique makes Tenon’s patch graft a procedure of choice in managing corneal perforations more so in peripheral corneal perforations in resource-limited health care facilities.

## Abbreviations

- AMG: Amniotic membrane graft
- TPG: Tenon’s patch graft
- ESR: Erythrocte sedimentation rate
- RBS: Random blood sugar
- VDRL: Venereal disease research laboratory test
- RA factor: Rheumatoid arthritis factor
- HCV: Hepatitis C virus
- SGPT: Serum glutamic pyruvic transaminase
- ANCA: Anti-neutrophillic cytoplasmic antibody
- ANA: Antinuclear antibodies
- HBsAg: Hepatitis B antigen

## Notes

### Competing interests

The authors declare that they have no competing interests.
